# Supplementary material for: Peatland organic matter quality varies with latitude as suggested by combination of FTIR and Ramped Pyrolysis Oxidation
Source: PLoS One. 2024 Nov 14;19(11):e0309654. doi: 10.1371/journal.pone.0309654 (PMC11563372; doi:10.1371/journal.pone.0309654)
Supplement: S1 File — Contains S1, S2 Figs and S1-S4 Tables. (DOCX) [file pone.0309654.s001.docx]

*Supporting information for*

Peatland organic matter quality varies with latitude as suggested by combination of FTIR and Ramped Pyrolysis Oxidation

Katy J. Sparrow^1,2*^, Jeffrey P. Chanton^1^, Ulrich M. Hanke^3,4^, Mark D. Kurz^3,4^, and Ann P. McNichol^5^

^1^Department of Earth, Ocean, and Atmospheric Science, Florida State University, Tallahassee, FL, United States of America

^2^Department of Geosciences, Georgia State University, Atlanta, GA, United States of America

^3^NOSAMS Laboratory, Geology and Geophysics, Woods Hole Oceanographic Institution, Woods Hole, MA, United States of America

^4^Marine Chemistry and Geochemistry, Woods Hole Oceanographic Institution, Woods Hole, MA, United States of America

^5^Geology and Geophysics, Woods Hole Oceanographic Institution, Woods Hole, MA, United States of America

*Corresponding author

E-mail: [ksparrow@gsu.edu](mailto:ksparrow@gsu.edu) (KJS)

**Contents of this file**

S1-S2 Figures

S1-S4 Tables

**Introduction**

Here, we present supplementary figures and data tables from the FTIR Spectroscopy analyses, Ramped Pyrolysis Oxidation (Ramped PyrOx), and carbon isotope preparations and analyses. The information for the numbered references cited within this supporting information file are found at the end of the document.

**S1 Figure.** Fourier-transform infrared spectroscopy (FTIR) absorbance spectra from **a)** Tropical, **b)** Subtropical, **c)** Boreal, and **d)** Polar peat cores. The absorbance from carbohydrate, aromatic, and aliphatic groups is indicated with red, black, and grey boxes, respectively. Mineral presence is indicated in two samples in panel **d)**, from absorption at 780 cm^-1^, which could have significantly increased absorption at 1030 cm^-1^, where absorption by carbohydrates occurs.

| 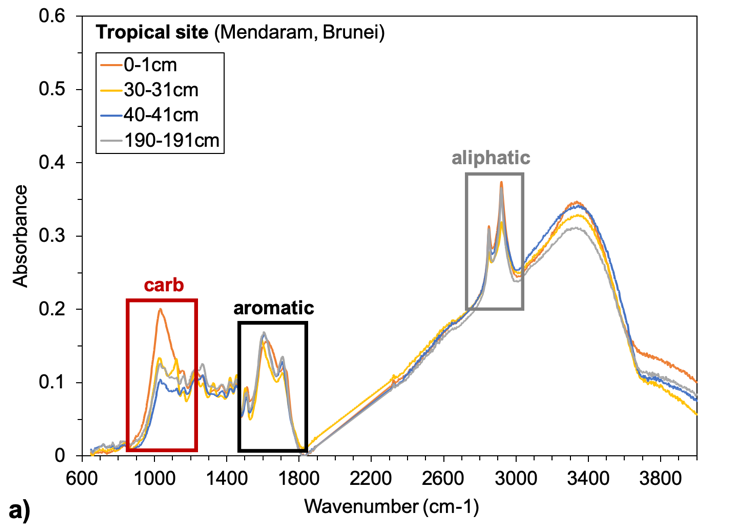 | 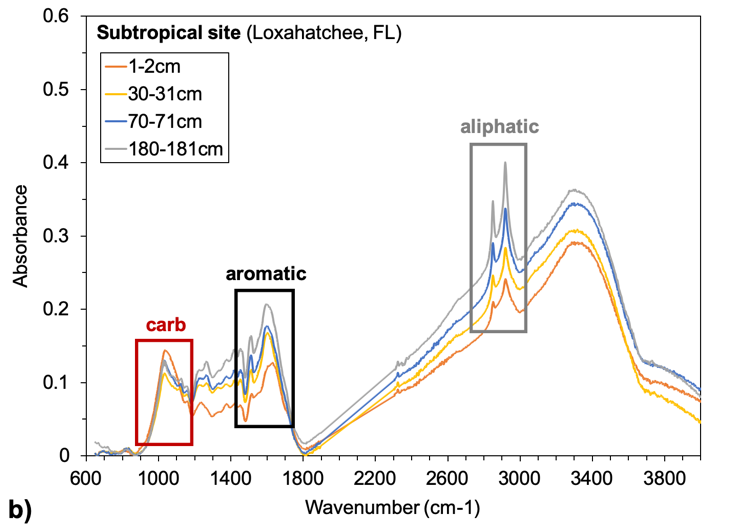 |
| --- | --- |
| 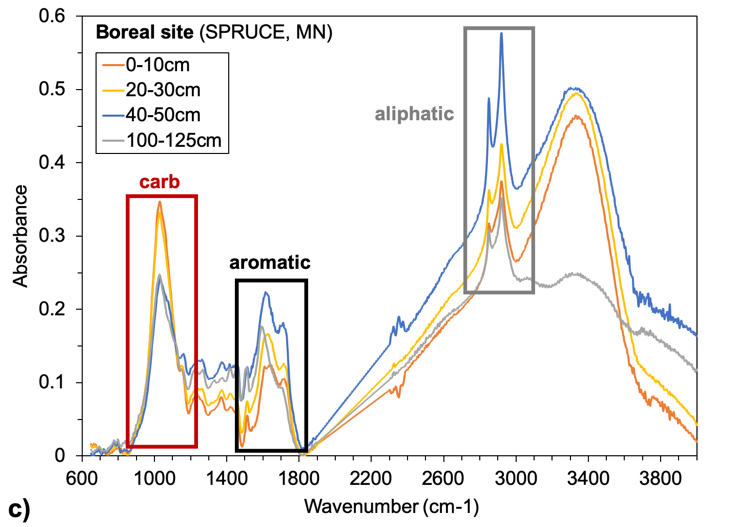 | 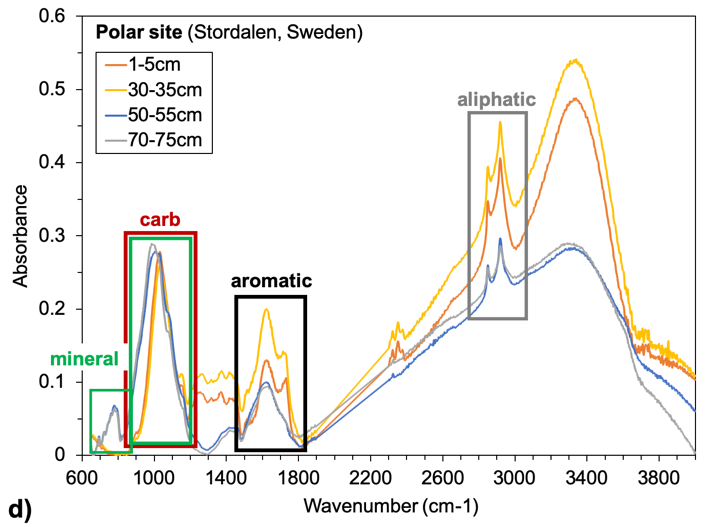 |

| **S2 Figure.** The estimated probability density functions (PDF) of activation energy “p(0,*E*)” for each of the 16 samples, organized by climate (columns) and depth (rows). PDF are outputs of the model described by Hemingway et al. [1, 2] after inputting the observed CO_2_ concentrations exiting from the heated sample oven and the measured temperature of the sample oven across the length of the sample preparation (resolution of 1 data point/ sec). The area under each Gaussian curve is 1. The x and y axes have the same range in each subplot.  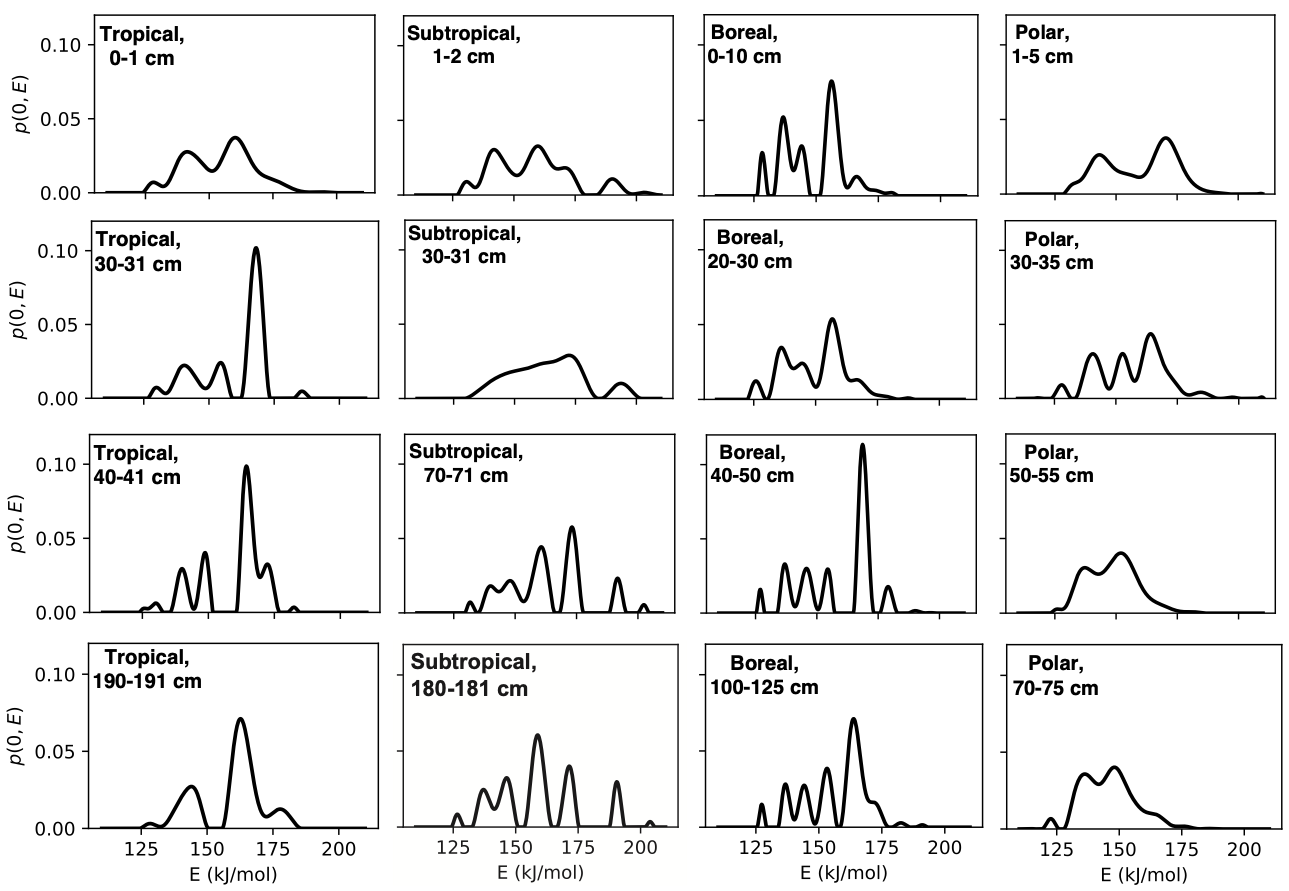  **S1 Table.**  Ramped PyrOx thermal windows for the splits collected from each sample and reference standard.   \| **Climate Type** \| **Site** \| **Sample depth (cm)** \| **Split 1 thermal window (°C)** \| **Split 2 thermal window (°C)** \| **Split 3 thermal window (°C)** \| **Split 4 thermal window (°C)** \| \| --- \| --- \| --- \| --- \| --- \| --- \| --- \| \| Tropical \| Mendaram \| 0-1 \| 229 - 326 \| 326 - 405 \| 405 - 518 \| - \| \| 30-31 \| 230 - 330 \| 330 - 437 \| 437 - 519 \| - \| \| 40-41 \| 232 - 329 \| 329 - 419 \| 419 - 504 \| - \| \| 190-191 \| 245 - 328 \| 328 - 415 \| 415 - 515 \| - \| \| Subtropical \| Loxahatchee \| 1-2 \| 220 - 374 \| 374 - 417 \| 417 - 481 \| 481 - 590 \| \| 30-31 \| 245 - 390 \| 390 - 453 \| 453 - 620 \| - \| \| 70-71 \| 247 - 347 \| 347 - 412 \| 412 - 469 \| 469 - 577 \| \| 180-181 \| - \| - \| - \| - \| \| Boreal \| Marcell Forest \| 0-10 \| 208 - 325 \| 325 - 390 \| 390 - 501 \| - \| \| 20-30 \| 200 - 304 \| 304 - 365 \| 365 - 400 \| 400 - 490 \| \| 40-50 \| 210 - 313 \| 313 - 382 \| 382 - 441 \| 441 - 530 \| \| 100-125 \| 215 - 314 \| 314 - 416 \| 416 - 514 \| - \| \| Polar \| Stordalen \| 1-5 \| 235 - 331 \| 331 - 436 \| 436 - 532 \| - \| \| 30-35 \| 206 - 323 \| 323 - 396 \| 396 - 439 \| 439 - 532 \| \| 50-55 \| 220 - 400 \| 400 - 480 \| - \| - \| \| 70-75 \| 193 - 306 \| 306 - 351 \| 351 - 485 \| - \| \| ^14^C Standards \| *NaHCO_3_ Fossil std \| - \| 101 - 190 \| 654 - 850 \| - \| - \| \| *NaHCO_3_ Modern std \| - \| 102 - 190 \| 654 - 850 \| - \| - \|   **S2 Table. a)** Radiocarbon (^14^C) blank correction calculations for the ^14^C signatures of the splits collected using the Ramped PyrOx method. ^14^C data were mass balance blank corrected [3] with some customization to account for the sequential thermal analysis [4]. **b)** Data for the internal standards that were prepared to perform the blank corrections. Modern and fossil sodium bicarbonate (NaHCO_3_) internal radiocarbon standards (n=2) were prepared during the sample preparation period in the same manner as the samples.  **a)**   \| **Location** \| \| **Mid-depth (cm)** \| **Split #** \| **SSL mass (µg C)** \| **Split temp (∆°C)** \| **Blank size (µg C)** \| **Fm_measured_** \| **uncertainty Fm_measured_** \| **Fm_corrected_** \| **uncertainty Fm_corrected_** \| \| --- \| --- \| --- \| --- \| --- \| --- \| --- \| --- \| --- \| --- \| --- \| \| Mendaram \| 0.5 \| \| 1 \| 101 \| 97 \| 2.6 \| 1.062 \| 0.003 \| **1.085** \| **0.007** \| \| Mendaram \| 0.5 \| \| 2 \| 153 \| 79 \| 2.2 \| 1.052 \| 0.004 \| **1.065** \| **0.005** \| \| Mendaram \| 0.5 \| \| 3 \| 102 \| 113 \| 3.0 \| 1.047 \| 0.003 \| **1.072** \| **0.007** \| \| Mendaram \| 30.5 \| \| 1 \| 139 \| 100 \| 2.7 \| 0.936 \| 0.003 \| **0.950** \| **0.005** \| \| Mendaram \| 30.5 \| \| 3 \| 133 \| 82 \| 2.3 \| 0.934 \| 0.003 \| **0.947** \| **0.005** \| \| Mendaram \| 40.5 \| \| 1 \| 152 \| 97 \| 2.6 \| 0.924 \| 0.002 \| **0.936** \| **0.004** \| \| Mendaram \| 40.5 \| \| 3 \| 234 \| 85 \| 2.3 \| 0.926 \| 0.002 \| **0.933** \| **0.003** \| \| Mendaram \| 190.5 \| \| 1 \| 111 \| 83 \| 2.3 \| 0.875 \| 0.002 \| **0.889** \| **0.004** \| \| Mendaram \| 190.5 \| \| 2 \| 261 \| 87 \| 2.4 \| 0.879 \| 0.002 \| **0.885** \| **0.003** \| \| Mendaram \| 190.5 \| \| 3 \| 175 \| 100 \| 2.7 \| 0.875 \| 0.002 \| **0.886** \| **0.004** \| \| Loxahatchee \| 1.5 \| \| 1 \| 349 \| 154 \| 4.0 \| 1.071 \| 0.003 \| **1.081** \| **0.004** \| \| Loxahatchee \| 1.5 \| \| 2 \| 159 \| 43 \| 1.4 \| 1.070 \| 0.003 \| **1.077** \| **0.004** \| \| Loxahatchee \| 1.5 \| \| 3 \| 138 \| 64 \| 1.9 \| 1.065 \| 0.003 \| **1.076** \| **0.004** \| \| Loxahatchee \| 1.5 \| \| 4 \| 63 \| 109 \| 2.9 \| 1.044 \| 0.003 \| **1.084** \| **0.011** \| \| Loxahatchee \| 70.5 \| \| 1 \| 149 \| 100 \| 2.7 \| 0.906 \| 0.002 \| **0.919** \| **0.004** \| \| Loxahatchee \| 70.5 \| \| 2 \| 201 \| 65 \| 1.9 \| 0.903 \| 0.002 \| **0.909** \| **0.003** \| \| Loxahatchee \| 70.5 \| \| 3 \| 168 \| 57 \| 1.7 \| 0.897 \| 0.002 \| **0.904** \| **0.003** \| \| Loxahatchee \| 70.5 \| \| 4 \| 89 \| 108 \| 2.9 \| 0.890 \| 0.003 \| **0.913** \| **0.007** \| \| Marcell Forest \| 5.0 \| \| 1 \| 85 \| 117 \| 3.1 \| 1.048 \| 0.003 \| **1.080** \| **0.008** \| \| Marcell Forest \| 5.0 \| \| 2 \| 85 \| 65 \| 1.9 \| 1.052 \| 0.004 \| **1.071** \| **0.006** \| \| Marcell Forest \| 5.0 \| \| 3 \| 52 \| 111 \| 3.0 \| 1.038 \| 0.004 \| **1.088** \| **0.012** \| \| Marcell Forest \| 25.0 \| \| 1 \| 111 \| 104 \| 2.8 \| 1.203 \| 0.003 \| **1.229** \| **0.006** \| \| Marcell Forest \| 25.0 \| \| 2 \| 157 \| 61 \| 1.8 \| 1.207 \| 0.004 \| **1.218** \| **0.005** \| \| Marcell Forest \| 25.0 \| \| 3 \| 76.2 \| 35 \| 1.2 \| 1.217 \| 0.004 \| **1.232** \| **0.005** \| \| Marcell Forest \| 25.0 \| \| 4 \| 86 \| 90 \| 2.5 \| 1.194 \| 0.003 \| **1.223** \| **0.007** \| \| Marcell Forest \| 45.0 \| \| 1 \| 126 \| 103 \| 2.8 \| 0.765 \| 0.002 \| **0.778** \| **0.003** \| \| Marcell Forest \| 45.0 \| \| 4 \| 155 \| 89 \| 2.4 \| 0.766 \| 0.003 \| **0.774** \| **0.004** \| \| Marcell Forest \| 112.5 \| \| 1 \| 127 \| 99 \| 2.7 \| 0.452 \| 0.002 \| **0.458** \| **0.002** \| \| Marcell Forest \| 112.5 \| \| 2 \| 378 \| 102 \| 2.7 \| 0.449 \| 0.002 \| **0.451** \| **0.002** \| \| Marcell Forest \| 112.5 \| \| 3 \| 194 \| 98 \| 2.6 \| 0.450 \| 0.002 \| **0.453** \| **0.002** \| \| Stordalen \| 3.0 \| \| 1 \| 76 \| 96 \| 2.6 \| 1.113 \| 0.003 \| **1.145** \| **0.009** \| \| Stordalen \| 3.0 \| \| 2 \| 171 \| 105 \| 2.8 \| 1.131 \| 0.003 \| **1.146** \| **0.005** \| \| Stordalen \| 3.0 \| \| 3 \| 81 \| 96 \| 2.6 \| 1.140 \| 0.004 \| **1.172** \| **0.009** \|   **S2 Table, continued**   \| **Location** \| \| **Mid-depth (cm)** \| **Split #** \| **SSL mass (µg C)** \| **Split temp (∆°C)** \| **Blank size (µg C)** \| **Fm_measured_** \| **uncertainty Fm_measured_** \| **Fm_corrected_** \| **uncertainty Fm_corrected_** \| \| --- \| --- \| --- \| --- \| --- \| --- \| --- \| --- \| --- \| --- \| --- \| \| Stordalen \| 32.5 \| \| 1 \| 243 \| 117 \| 3.1 \| 0.967 \| 0.003 \| **0.977** \| **0.004** \| \| Stordalen \| 32.5 \| \| 2 \| 396 \| 73 \| 2.1 \| 0.968 \| 0.003 \| **0.972** \| **0.003** \| \| Stordalen \| 32.5 \| \| 3 \| 258 \| 43 \| 1.4 \| 0.970 \| 0.003 \| **0.974** \| **0.003** \| \| Stordalen \| 32.5 \| \| 4 \| 154 \| 93 \| 2.5 \| 0.972 \| 0.003 \| **0.985** \| **0.004** \| \| Stordalen \| 72.5 \| \| 1 \| 215 \| 113 \| 3.0 \| 0.684 \| 0.002 \| **0.691** \| **0.003** \| \| Stordalen \| 72.5 \| \| 2 \| 240 \| 45 \| 1.4 \| 0.683 \| 0.002 \| **0.686** \| **0.002** \| \| Stordalen \| 72.5 \| \| 3 \| 241 \| 134 \| 3.5 \| 0.684 \| 0.002 \| **0.691** \| **0.002** \|   **b)**   \| **ID** \| **Split #** \| **^14^C**  **(Fm)** \| **^14^C Age**  **(yr BP)** \| **δ^13^C (‰)** \| \| --- \| --- \| --- \| --- \| --- \| \| NaHCO_3_ ^14^C_depleted_  (Fm = 0.0119±0.0013; δ^13^C = -5.7 ‰) \| 1 \| 0.009 \| 38,000 \| -6.22 \| \| 2 \| 0.021 \| 30,900 \| -6.68 \| \| NaHCO_3_ ^14^C_modern_  (Fm = 1.0354±0.0021; δ^13^C = -4.2 ‰) \| 1 \| 1.026 \| >Modern \| -4.37 \| \| 2 \| 1.018 \| >Modern \| -5.05 \|   **S3 Table.** Ramped PyrOx data was input to the model described by Hemingway et al. [1, 2], which uses the observed amount of CO_2_ evolving at each temperature across time to estimate the activation energy (*E*) required to break the many carbon bonds in each peat sample. The resulting probability density function (PDF) of required *E* was divided into three fractions: *E* < 150 kJ/mol “low” energy, 150 ≤ *E* ≤ 175 kJ/mol “medium” energy, and *E* > 175 kJ/mol “high” energy. The table shows each sample’s PDF binned into these three fractions; each row in the table sums to 100%.   \|  \|  \| **Percent of sample occurring in each activation energy category** \| \| \| \| --- \| --- \| --- \| --- \| --- \| \|  \| **Peat sample depth**  **(cm)** \| *E* < 150 kJ/mol “low”  (%) \| 150 ≤ *E* ≤ 175 kJ/mol  “medium”  (%) \| *E* > 175 kJ/mol “high”  (%) \| \|  \| \| **Tropical** \| 0-1 \| 39 \| 56 \| 5 \| \| 30-31 \| 25 \| 73 \| 2 \| \| 40-41 \| 30 \| 69 \| 1 \| \| 190-191 \| 28 \| 65 \| 7 \| \| **Subtropical** \| 1-2 \| 36 \| 54 \| 10 \| \| 30-31 \| 21 \| 62 \| 17 \| \| 70-71 \| 24.5 \| 63.5 \| 12 \| \| 180-181 \| 35 \| 54 \| 11 \| \| **Boreal** \| 0-10 \| 47 \| 52 \| 1 \| \| 20-30 \| 46 \| 53 \| 1 \| \| 40-50 \| 32 \| 60 \| 8 \| \| 100-125 \| 27 \| 71 \| 2 \| \| **Polar** \| 1-5 \| 33 \| 56 \| 11 \| \| 30-35 \| 32 \| 63 \| 5 \| \| 50-55 \| 54.5 \| 44.5 \| 1 \| \| 70-75 \| 67 \| 32 \| 1 \|   **S4 Table.** Radiocarbon (^14^C) and stable carbon isotope (δ^13^C) data reported for each sample (“bulk”) and for sequential splits collected from separate preparations of those same samples using Ramped PyrOx; some collected splits were not analyzed for ^14^C and/or δ^13^C. The ^14^C values and ages shown for the Ramped PyrOx splits are blank-corrected (**S2 Table**). Data is organized by climate: **a)** Tropical, **b)** Subtropical**, c)** Boreal, and **d)** Polar. ^14^C content is reported as Fraction Modern (Fm) and ^14^C age is reported in years before present (yr BP), where present is defined as 1950 C.E. [5]. δ^13^C data is reported in permille notation (‰).   1. **Tropical peat (Ulu Mendaram Conservation Area, Brunei; Core ID: MDM 11-2A)**  \| **Depth, midpoint**  **(cm)** \| **Bulk or Split #** \| **^14^C (Fm)** \| **^14^C age (yr BP)** \| **δ^13^C (‰)** \| \| --- \| --- \| --- \| --- \| --- \| \| 0.5 \| bulk \| 1.061 \| >Modern \| -30.32 \| \| 1 \| 1.085 \| >Modern \| nd \| \| 2 \| 1.065 \| >Modern \| -31.36 \| \| 3 \| 1.072 \| >Modern \| -30.14 \| \| 30.5 \| bulk \| 0.938 \| 515 \| -29.31 \| \| 1 \| 0.950 \| 410 \| -29.82 \| \| 3 \| 0.947 \| 442 \| -28.77 \| \| 40.5 \| bulk \| 0.929 \| 590 \| -29.88 \| \| 1 \| 0.936 \| 529 \| -30.95 \| \| 3 \| 0.933 \| 553 \| -29.04 \| \| 190.5 \| bulk \| 0.882 \| 1,010 \| -30.36 \| \| 1 \| 0.889 \| 942 \| -30.66 \| \| 2 \| 0.885 \| 984 \| -30.64 \| \| 3 \| 0.886 \| 974 \| -29.20 \| |
| --- | --- | --- | --- | --- | --- | --- | --- | --- | --- | --- | --- | --- | --- | --- | --- | --- | --- | --- | --- | --- | --- | --- | --- | --- | --- | --- | --- | --- | --- | --- | --- | --- | --- | --- | --- | --- | --- | --- | --- | --- | --- | --- | --- | --- | --- | --- | --- | --- | --- | --- | --- | --- | --- | --- | --- | --- | --- | --- | --- | --- | --- | --- | --- | --- | --- | --- | --- | --- | --- | --- | --- | --- | --- | --- | --- | --- | --- | --- | --- | --- | --- | --- | --- | --- | --- | --- | --- | --- | --- | --- | --- | --- | --- | --- | --- | --- | --- | --- | --- | --- | --- | --- | --- | --- | --- | --- | --- | --- | --- | --- | --- | --- | --- | --- | --- | --- | --- | --- | --- | --- | --- | --- | --- | --- | --- | --- | --- | --- | --- | --- | --- | --- | --- | --- | --- | --- | --- | --- | --- | --- | --- | --- | --- | --- | --- | --- | --- | --- | --- | --- | --- | --- | --- | --- | --- | --- | --- | --- | --- | --- | --- | --- | --- | --- | --- | --- | --- | --- | --- | --- | --- | --- | --- | --- | --- | --- | --- | --- | --- | --- | --- | --- | --- | --- | --- | --- | --- | --- | --- | --- | --- | --- | --- | --- | --- | --- | --- | --- | --- | --- | --- | --- | --- | --- | --- | --- | --- | --- | --- | --- | --- | --- | --- | --- | --- | --- | --- | --- | --- | --- | --- | --- | --- | --- | --- | --- | --- | --- | --- | --- | --- | --- | --- | --- | --- | --- | --- | --- | --- | --- | --- | --- | --- | --- | --- | --- | --- | --- | --- | --- | --- | --- | --- | --- | --- | --- | --- | --- | --- | --- | --- | --- | --- | --- | --- | --- | --- | --- | --- | --- | --- | --- | --- | --- | --- | --- | --- | --- | --- | --- | --- | --- | --- | --- | --- | --- | --- | --- | --- | --- | --- | --- | --- | --- | --- | --- | --- | --- | --- | --- | --- | --- | --- | --- | --- | --- | --- | --- | --- | --- | --- | --- | --- | --- | --- | --- | --- | --- | --- | --- | --- | --- | --- | --- | --- | --- | --- | --- | --- | --- | --- | --- | --- | --- | --- | --- | --- | --- | --- | --- | --- | --- | --- | --- | --- | --- | --- | --- | --- | --- | --- | --- | --- | --- | --- | --- | --- | --- | --- | --- | --- | --- | --- | --- | --- | --- | --- | --- | --- | --- | --- | --- | --- | --- | --- | --- | --- | --- | --- | --- | --- | --- | --- | --- | --- | --- | --- | --- | --- | --- | --- | --- | --- | --- | --- | --- | --- | --- | --- | --- | --- | --- | --- | --- | --- | --- | --- | --- | --- | --- | --- | --- | --- | --- | --- | --- | --- | --- | --- | --- | --- | --- | --- | --- | --- | --- | --- | --- | --- | --- | --- | --- | --- | --- | --- | --- | --- | --- | --- | --- | --- | --- | --- | --- | --- | --- | --- | --- | --- | --- | --- | --- | --- | --- | --- | --- | --- | --- | --- | --- | --- | --- | --- | --- | --- | --- | --- | --- | --- | --- | --- | --- | --- | --- | --- | --- | --- | --- | --- | --- | --- | --- | --- | --- | --- | --- | --- | --- | --- | --- | --- | --- | --- | --- | --- | --- | --- | --- | --- | --- | --- | --- | --- | --- | --- | --- | --- | --- | --- | --- | --- | --- | --- | --- | --- | --- | --- | --- | --- | --- | --- | --- | --- | --- | --- | --- | --- | --- | --- | --- | --- | --- | --- | --- | --- | --- | --- | --- | --- | --- | --- | --- | --- | --- | --- | --- | --- | --- | --- | --- | --- | --- | --- | --- | --- | --- | --- | --- | --- | --- | --- | --- | --- | --- | --- | --- | --- | --- | --- | --- | --- | --- | --- | --- | --- | --- | --- | --- | --- | --- | --- | --- | --- | --- | --- | --- | --- | --- | --- | --- | --- | --- | --- | --- | --- | --- | --- | --- | --- | --- | --- | --- | --- | --- | --- | --- | --- | --- | --- | --- | --- | --- | --- | --- | --- | --- | --- | --- | --- | --- | --- | --- | --- | --- | --- | --- | --- | --- | --- | --- | --- | --- | --- | --- | --- | --- | --- | --- | --- | --- | --- | --- | --- | --- | --- | --- | --- | --- | --- | --- | --- | --- | --- | --- | --- | --- | --- | --- | --- | --- | --- | --- | --- | --- | --- | --- | --- | --- | --- | --- | --- | --- | --- | --- | --- | --- | --- | --- | --- | --- | --- | --- | --- | --- | --- | --- | --- | --- | --- | --- | --- | --- | --- | --- | --- | --- | --- | --- | --- | --- | --- | --- | --- | --- | --- | --- | --- | --- | --- | --- | --- | --- | --- | --- | --- | --- | --- | --- | --- | --- | --- | --- | --- | --- | --- | --- | --- | --- | --- | --- | --- | --- | --- | --- | --- | --- | --- |

1. **Subtropical peat (Loxahatchee National Wildlife Refuge, Florida, USA; Core ID: Lox3)**

*Data reported by [6]

| **Depth, midpoint**  **(cm)** | **Bulk or Split #** | **^14^C (Fm)** | **^14^C age (yr BP)** | **δ^13^C (‰)** |
| --- | --- | --- | --- | --- |
| 1.5 | bulk | 1.074 | >Modern | -27.37 |
|  | 1 | 1.081 | >Modern | -28.83 |
|  | 2 | 1.077 | >Modern | -28.49 |
|  | 3 | 1.076 | >Modern | -28.08 |
|  | 4 | 1.084 | >Modern | -28.34 |
| 30.5 | bulk | 0.972 | 225 | -25.11 |
| 70.5 | bulk | 0.901 | 835 | -26.33 |
|  | 1 | 0.919 | 678 | nd |
|  | 2 | 0.909 | 763 | -26.85 |
|  | 3 | 0.904 | 814 | -26.72 |
|  | 4 | 0.913 | 728 | -26.67 |
| 180.5 | *bulk | 0.747 | 2,340 | nd |

**S4 Table, continued**

1. **Boreal peat (Marcell Experimental Forest, Minnesota, USA; Core ID: T3F)**

| **Depth, midpoint**  **(cm)** | **Bulk or Split #** | **^14^C (Fm)** | **^14^C age (yr BP)** | **δ^13^C (‰)** |
| --- | --- | --- | --- | --- |
| 5 | bulk | 1.066 | >Modern | -29.64 |
|  | 1 | 1.080 | >Modern | -30.58 |
|  | 2 | 1.071 | >Modern | -30.85 |
|  | 3 | 1.088 | >Modern | -29.95 |
| 25 | bulk | 1.214 | >Modern | -27.24 |
|  | 1 | 1.229 | >Modern | -27.29 |
|  | 2 | 1.218 | >Modern | -28.30 |
|  | 3 | 1.232 | >Modern | nd |
|  | 4 | 1.223 | >Modern | -27.34 |
| 45 | bulk | 0.773 | 2,070 | -26.63 |
|  | 1 | 0.778 | 2,019 | -27.08 |
|  | 4 | 0.774 | 2,053 | -25.90 |
| 112.5 | bulk | 0.459 | 6,250 | -27.31 |
|  | 1 | 0.458 | 6,281 | -27.25 |
|  | 2 | 0.451 | 6,401 | -28.06 |
|  | 3 | 0.453 | 6,360 | -26.97 |

1. **Polar peat (Stordalen Mire, Sweden; Core ID: CPP)**

| **Depth, midpoint**  **(cm)** | **Bulk or Split #** | **^14^C (Fm)** | **^14^C age (yr BP)** | **δ^13^C (‰)** |
| --- | --- | --- | --- | --- |
| 3 | bulk | 1.114 | >Modern | -26.31 |
|  | 1 | 1.145 | >Modern | -27.06 |
|  | 2 | 1.146 | >Modern | -28.98 |
|  | 3 | 1.172 | >Modern | -27.64 |
| 32.5 | bulk | 0.970 | 245 | -25.80 |
|  | 1 | 0.977 | 186 | -26.61 |
|  | 2 | 0.972 | 229 | -27.63 |
|  | 3 | 0.974 | 214 | -26.89 |
|  | 4 | 0.985 | 125 | -26.27 |
| 52.5 | bulk | 0.730 | 2,530 | -28.36 |
| 72.5 | bulk | 0.686 | 3,030 | -28.30 |
|  | 1 | 0.691 | 2,972 | -29.09 |
|  | 2 | 0.686 | 3,031 | -28.99 |
|  | 3 | 0.691 | 2,975 | -28.22 |

**References**

1. Hemingway JD, Rothman DH, Rosengard SZ, Galy VV. Technical note: An inverse method to relate organic carbon reactivity to isotope composition from serial oxidation. Biogeosciences. 2017. doi:10.5194/bg-14-5099-2017.
2. Hemingway JD. *rampedpyrox*: open-source tools for thermoanalytical data analysis (1.0.3) [Software]. <http://pypi.python.org/pypi/rampedpyrox> [online, accessed 2020 May 1].
3. Hanke UM, Wacker L, Haghipour N, Schmidt MWI, Eglinton TI, McIntyre CP. Comprehensive radiocarbon analysis of benzene polycarboxylic acids (BPCAs) derived from pyrogenic carbon in environmental samples. Radiocarbon. 2017. doi:10.1017/RDC.2017.44.
4. Hanke UM, Gagnon AR, Reddy CM, Lardie Gaylord MC, Cruz AJ, Galy V, et al. Sequential thermal analysis of complex organic mixtures: Procedural standards and improved CO_2_ purification capacity. Radiocarbon. 2023. doi:10.1017/RDC.2023.13.
5. Stuiver M, Polach HA. Discussion reporting of 14C data. Radiocarbon. 1977. doi:10.1017/S0033822200003672.
6. Hodgkins SB, Richardson CJ, Dommain R, Wang H, Glaser PH, Verbeke B, et al. Tropical peatland carbon storage linked to global latitudinal trends in peat recalcitrance. Nat Commun. 2018. doi:10.1038/s41467-018-06050-2.
