# Supplementary material for: Peatland organic matter quality varies with latitude as suggested by combination of FTIR and Ramped Pyrolysis Oxidation
Source: PLoS One. 2024 Nov 14;19(11):e0309654. doi: 10.1371/journal.pone.0309654 (PMC11563372; doi:10.1371/journal.pone.0309654)
Supplement: Attachment — Submitted filename: Response to Reviewers.docx [file pone.0309654.s002.docx]

Journal Requirements:

When submitting your revision, we need you to address these additional requirements.

1. Please ensure that your manuscript meets PLOS ONE's style requirements, including those for file naming. The PLOS ONE style templates can be found at

<https://journals.plos.org/plosone/s/file?id=wjVg/PLOSOne_formatting_sample_main_body.pdf> and

<https://journals.plos.org/plosone/s/file?id=ba62/PLOSOne_formatting_sample_title_authors_affiliations.pdf>

Done.

2.In your Methods section, please provide additional information regarding the permits you obtained for the work. Please ensure you have included the full name of the authority that approved the field site access and, if no permits were required, a brief statement explaining why.

Now included in Methods section:

“No permits were required to access or perform work in the field sites.”

3. Thank you for stating the following financial disclosure:

"KJS was supported by a Diversity Postdoctoral Scholar Award from the Provost’s Office at Florida State University. KJS and JPC were awarded a Radiocarbon Research Initiative by the National Ocean Sciences Accelerator Mass Spectrometer Facility. "

Please state what role the funders took in the study.  If the funders had no role, please state: "The funders had no role in study design, data collection and analysis, decision to publish, or preparation of the manuscript."

If this statement is not correct you must amend it as needed.

Please include this amended Role of Funder statement in your cover letter; we will change the online submission form on your behalf.

Our amended Role of Funder statement, included in our cover letter:

"KJS was supported by a Diversity Postdoctoral Scholar Award from the Provost’s Office at Florida State University (FSU). KJS and JPC were awarded a Radiocarbon Research Initiative by the National Ocean Sciences Accelerator Mass Spectrometer Facility (NOSAMS). UMH was supported by the National Science Foundation (NSF) during his postdoctoral position at NOSAMS (OCE 1755125). Neither the Provost’s Office at FSU nor NSF had a role in study design, data collection and analysis, decision to publish, or preparation of the manuscript. Laboratory preparations and analyses were performed at NOSAMS by the authors. Two co-authors (UMH and MDK) are affiliated with NOSAMS.”

Additional Editor Comments:

I think that this paper should be major revision before publication.

Reviewer #1: General comments

The authors introduce research towards use of FTIR and the Ramped PyrOx method for assessing the quality of organic matter content of peatlands situated in four different latitudes and climates. The paper is very well-written and is clearly understandable throughout. The Methodology is detailed and mostly adequately justified. Reporting of results is sequential and clear. However, I recommend some major considerations.

Firstly, the aim of this work is not well justified and feels inconsistent throughout the paper. Initially, the paper reads as work towards replicating a finding (latitude vs OM quality trends) using another method. Whilst this in and of itself is legitimate work, the justification of why this work is necessary is not clear from the outset. As the paper progresses, more emphasis is placed on how these methods can be used in combination to help build a more complete picture of peat formation and OM quality at a site, as well as an exploration of which methods are appropriate/inappropriate for particular sites or contexts. This is well explored in the Discussion and, as a scientist with an interest in peat carbon, this felt like the most interesting part of the work to me. As such, I would suggest that the authors rework their Abstract, Aim and Introduction to better reflect the content of the Discussion. I am happy for authors to disagree with my suggestion to change the scope of their aim, but whatever the authors decide going forward, I think that a reworking of the paper is necessary so that a clear and well-justified aim is a constant thread throughout the text. This would help to make the purpose and value of the work more clear.

Thank you for this critique. We have adjusted the Abstract, Aim, and Introduction per your suggestion so that these sections are consistent with our Discussion.

Secondly, I would be interested to know why more cores from each site were not sampled for Ramped PyrOx analysis. The lack of replicates for each depth section at each of the sites could have granted you more statistical power to investigate how methods compare within sites at different latitudes. Much research points towards the heterogeneity of peat carbon stocks within individual peatland systems, and the inclusion of more samples could also help you to better characterise the range of values found at different latitudes. If more samples cannot be included in a revised version, the lack of replicates at each site needs to be more thoroughly addressed as a limitation in the Discussion, and how this limits any interpretations you make.

In the revision, we do more to address the small sample size and lack of core replicates at each site as a limitation and use language that better acknowledges how this limits our study’s interpretations.

“The small sample set studied herein and lack of core replicates presents a significant limitation of this study.”

The relatively small number of samples in our study is due to our study employing the Ramped PyrOx technique, which requires several hours to process one sample or standard. Due to great demand on this unique, in-demand instrumentation in the NOSAMS sample preparation laboratory, there is limited access to any one user.

As made clearer in the revised ms, the sample set examined with three methods in this study is a small subset of the much larger studies of Hodgkins et al. 2018 and Verbeke et al. 2022, which utilized FTIR only. Both of these prior studies that set the context for our study analyzed multiple cores at the majority of their sites. With 14 cores from seven distinct peatland sites, Hodgkins et al. found a clear trend of peat composition varying distance from the equator, with latitude/ climate acting as master variables dictating peat composition and accumulation. Verbeke et al. vastly expanded upon the work of Hodgkins et al. by analyzing over 1,000 samples from 165 peatland sites, including the sites in our study, or in close proximity to them. A key data figure from each study is shown below and here is some additional text now found in our Materials and Methods section under “Sites and sampling”:

“Hodgkins et al. [27] analyzed two cores at our Tropical site, two cores at our Subtropical site, two cores at our Boreal site, and one core at our Polar site. Verbeke et al. [30] analyzed eight cores at our Tropical site, no cores at our Subtropical site, but three cores located within one degree of latitude south of our site at another Everglades location, 21 cores at our Boreal site, and eight cores at our Polar site.”

|  | **Figure 3** reproduced from Hodgkins et al. (2018), *Nature Communications*, shows strong correlations found through linear regression analysis between peat carbohydrates/ aromatics content and latitude (**a, b**) and mean annual temperature (**c, d**) when analyzing multiple cores at several sites; in this figure, the Minnesota bogs and Minnesota fens are grouped together. Each data point represents the average ± one standard deviation of core sections within the top 50 cm of each core. |
| --- | --- |
|  | **Figure 3 a, b** reproduced from Verbeke et al. (2022), *Global Biogeochemical Cycles*, shows through linear mixed modeling that peat carbohydrate content increased from the equator toward the poles as peat aromatics content decreased. |

Thirdly, no figures were available to review for the paper - only supplementary figures within the PDF were supplied to reviewers. The next version of the paper should include copies of these figures so that these can be peer reviewed too - the Results and Discussion were difficult to digest without visual aids. I am unsure whether this was an issue with the PLOS ONE editorial system and their distribution of materials to reviewers, or an issue with submission at the authors’ end. Regardless, I hope this can be resolved.

All seven figures are found in the file inventory for the original manuscript submission, so, from our end, we do not know any reason why they were not available to you. All figures were submitted separate from, not embedded within, the uploaded manuscript file, per journal requirements.

We value your review of the figures and to ensure that you can view them, we have included the revised figures at the end of this ‘Response to Reviewers’ file.

I recommend that this paper is revised with major corrections based on the above feedback and my specific comments below. I hope the authors find the comments constructive to help improve the impact of their work.

Specific comments

Introduction

L44: replace “gases” with “gas”.

Done

It is not clear what the value of Ramped PyrOx as a method is from the Introduction. Some elaboration on the benefits of the Ramped PyrOx method (both as standalone and compared to FTIR/other methods) would be useful.

Done

Study site and methods

A description of statistical methods used for comparison between methods is needed in this section.

We used simple linear regression to compare the two methods. We have included this info in the newly created “Method Comparison” part of the Results section (in response to the reviewer’s next suggestion, below), as few details are needed.

“To evaluate H1, the hypothesis that the Ramped PyrOx approach of evaluating OM quality would be consistent with that of FTIR, we examined the correlations among the data stemming from these two methods using simple linear regression. Three statistically significant correlations were observed from linear regression analysis between the estimated abundance of three molecular functional groups determined from FTIR (area-normalized peak heights of carbohydrates, aromatics, and aliphatics) and the estimated fraction of each sample occurring in the three defined E categories determined from Ramped PyrOx (Fig 7, Table 2).”

Discussion

The key results which pertain to the aim of this paper are being reported in the Discussion – these should be moved to the Results section, and any interpretation of these results should remain in the Discussion.

Done.

L442: replace “intense” with “intensive”.

Done

**Fig 1.** **Elemental analysis.** Elemental analyses were conducted to determine a) the Carbon content (%C), b) Nitrogen content (%N), and c) Carbon to Nitrogen ratios (C:N) in the Tropical (orange), Subtropical (yellow), Boreal (green), and Polar (purple) peat cores.

**Fig 2.** **FTIR depth profiles.** Depth profiles for a) Carbohydrate, b) Aromatics, and c) Aliphatics functional groups in Tropical (orange), Subtropical (yellow), Boreal (green), and Polar (purple) peat cores (n=16). Data shown are the peak heights measured with FTIR normalized to the total area of the spectrum. In a), the open circles of the deepest two Polar peat samples indicate that these measurements were likely affected (inflated) by the presence of minerals which absorb in the same area of the IR spectrum as carbohydrates.

**Fig 3. Ramped PyrOx thermographs.** Thermographs show how CO_2_ evolved from each combusting peat sample at an oven ramp rate of +5˚C/ minute as well as the ^14^C measurements of the temperature-separated CO_2_ fractions. Peat core data from a) Tropical, b) Subtropical, c) Boreal, and d) Polar climates are shown. These results are presented as function of the temperature of combustion for each serially oxidized sample. The blank-corrected ^14^C content of individual temperature-separated “splits” or fractions of a single sample are shown as diamonds with horizontal lines to indicate the temperature range across which each split was formed; the total process ^14^C error is smaller than the markers. The CO_2_ that evolved as the sample was combusted at increasing temperature is normalized to the total amount of CO_2_ that evolved during the sample preparation, so the area under each Gaussian curve is equal to 1. Asterisks (*) superimposed on the normalized pCO_2_ data indicate the start/ stop of the splits that were collected for ^14^C analyses. The x and y axes have the same range in each subplot.

**Fig 4. Carbon reactivity depth profiles.** Depth profiles for each peat core showing what proportion of Carbon (C) in each sample was observed to occur in a) low (*E* <150 kJ/mol), b) medium (150 ≤ *E* ≤ 175 kJ/mol), and c) high (*E* >175 kJ/mol) categories of activation energy (*E*). The fraction of C occurring in each of the three defined *E* categories was quantified by calculating the area under the curve in each *E* category in the estimated probability density functions (**S2 Fig**). Data is shown for the Tropical (orange), Subtropical (yellow), Boreal (green), and Polar (purple) peat cores (n=16).

**Fig 5. First derivative of the estimated probability density function of the activation energy required to break the Carbon bonds in each sample.** The cumulative area under the p(0,*E*) curve as a function of activation energy (*E*) for each sample depth in the a) Tropical, b) Subtropical, c) Boreal, and d) Polar peat cores. Boundaries for the three activation energy categories are represented by the vertical dashed lines. Where the data has no slope, no carbon exists at those values of *E* and, conversely, across values of *E* where the data has a near-vertical slope, there are relatively large amounts of carbon.

**Fig 6. Radiocarbon content of Ramped PyrOx splits compared to bulk samples.** The radiocarbon (^14^C) content of the temperature- separated fractions (“splits”) collected from the Ramped PyrOx method (diamonds) were observed to be equivalent to the ^14^C content of the bulk sample (dashed lines). In many instances, the Ramped PyrOx splits revealed such a high homogeneity in ^14^C that they appear as one marker. Error bars are smaller than the markers.

**Fig 7.** **Linear regressions comparing FTIR and Ramped PyrOx data.** Statistically significant relationships between normalized carbohydrate and aromatic peak heights (FTIR) and the proportion of each sample occurring in three defined categories of activation energy (*E*; Ramped PyrOx). a) Positive correlation between carbohydrates and the “low” *E* fraction, excluding two samples affected by mineral interference (open circles), b) Negative correlation between aromatics and the “low” *E* fraction, c) Positive correlation between aromatics and the “medium” *E* fraction. Data is shown for the Tropical (orange), Subtropical (yellow), Boreal (green), and Polar (purple) peat cores (n=16).
